# Lnc-ing Trained Immunity to Chromatin Architecture

**DOI:** 10.3389/fcell.2019.00002

**Published:** 2019-01-24

**Authors:** Stephanie Fanucchi, Musa M. Mhlanga

**Affiliations:** ^1^Division of Chemical, Systems & Synthetic Biology, Department of Integrative Biomedical Sciences, Faculty of Health Sciences, Institute of Infectious Disease & Molecular Medicine, University of Cape Town, Cape Town, South Africa; ^2^Gene Expression and Biophysics Group, CSIR Biosciences, Pretoria, South Africa

**Keywords:** LncRNA—long non-coding RNA, chromatin, trained immunity, transcriptional (regulation), innate immunity, epigenetic memory, macrophages, BCG—Bacille Calmette-Guérin vaccine

## Abstract

Human innate immune cells exposed to certain infections or stimuli develop enhanced immune responses upon re-infection with a different second stimulus, a process termed trained immunity. Recent studies have revealed that hematopoietic stem cells (HSCs) are integral to trained immune responses as they are able to “remember” transcriptional responses and transmit this state to their progeny to educate them how to respond to future infections. The macrophages that arise from trained HSCs are epigenetically reprogrammed and as a result robustly express immune genes, enhancing their capability to resolve infection. Accumulation of H3K4me3 epigenetic marks on multiple immune gene promoters underlie robust transcriptional responses during trained immune responses. However, the mechanism underpinning how these epigenetic marks accumulate at discrete immune gene loci has been poorly understood. In this review, we discuss the previously unexplored contributions of nuclear architecture and long non-coding RNAs on H3K4me3 promoter priming in trained immunity. Altering the activity of these lncRNAs presents a promising therapeutic approach to achieve immunomodulation in inflammatory disease states.

By sensing external stimuli and launching a transcriptional program to resolve the infectious agent, innate immune responses are often sufficient to eradicate pathogens. Historically, innate immune cells have been thought to lack immunological memory, with only cells of the adaptive immune system being responsible for specificity and memory. However, numerous studies have challenged the view that innate immunity has no memory, most significantly with the emergence of the concept of trained immunity or innate immune memory.

Early studies observed that infection with a Candida species in mice lacking T and B cells offered protection from reinfection in a macrophage-dependent manner, although how this occurred remained unknown (Bistoni et al., 1986). These early observations are strongly supported by more recent studies which also show that monocytes and macrophages pre-exposed to certain stimuli (e.g., β-glucan) prior to exposure to certain infectious agents, acquired a memory of this exposure leading to enhanced resistance upon reinfection with a second infectious agent (Kleinnijenhuis et al., 2012; Quintin et al., 2012; Saeed et al., 2014; Netea et al., 2016). This response, shown to occur in the absence of key adaptive immune cells (T and B cells) is referred to as innate immune memory or “trained immunity.” The enhanced immunity observed in trained monocytes and macrophages is driven by epigenetic modifications that modulate the expression of innate immune genes (Quintin et al., 2012).

## Hematopoietic Stem Cells (HSCs) are Essential to Trained Immunity

**Figure 1:**
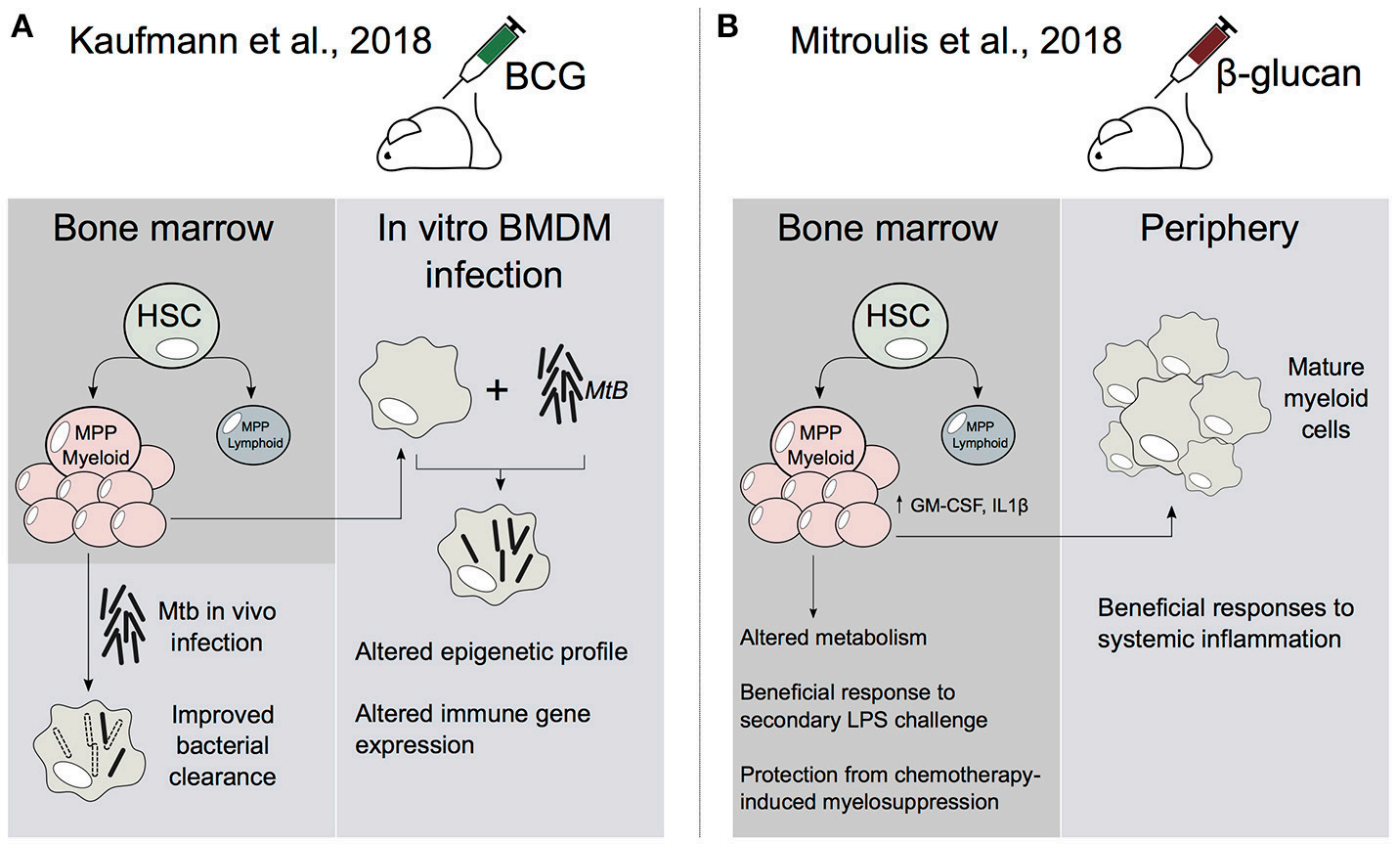
HSCs are central to the establishment of trained immunity. **(A)** In Kaufmann et al., BCG alters the transcriptome profile of HSC and multipotent progenitors (MPP). This leads to an increase in myelopoiesis which generates epigenetically reprogrammed monocytes and macrophages that are enhanced in their ability to resolve Mtb infection. **(B)** In Mitroulis et al., β-glucan induces the expansion of HSCs and MPPs. In addition, β-glucan altered IL1β and GM-CSF signaling as well as glucose and lipid metabolism in MPPs. Peripheral myeloid cells derived from β-glucan-trained progenitors were enhanced in their ability to resolve systemic inflammation.

Innate immune memory has mostly been studied in circulating monocytes and macrophages that are derived from the bone marrow (BM). In general, circulating monocytes have a relatively short lifespan and limited proliferative capacity. Therefore, it has been unclear how circulating monocytes and macrophages transmit trained immune memory to aid the persistence of the trained response for longer time periods, even the lifespan of the organism. Recent studies have revealed that the hematopoietic stem cells (HSCs) are integral to trained immunity (Figure 1, Kaufmann et al., 2018; Mitroulis et al., 2018). In adults, HSCs are located in the BM, and give rise to progenitor cells which ultimately differentiate into all lineages of mature blood cells, such as macrophages, monocytes, and dendritic cells (Cabezas-Wallscheid et al., 2014). As HSCs persist for the lifetime of an organism, adult HSCs exist predominantly in a quiescent state. Yet, HSCs retain the ability to dynamically exit quiescence and rapidly proliferate in response to environmental factors and stimuli such as infection. Importantly, it has been shown that HSCs are able to respond to pathogens through pattern-recognition receptors (PRRs) as well as cytokine and growth factors by activating a transcriptional network that may bias HSCs toward either the myeloid or lymphoid lineages (King and Goodell, 2011).

Bacille Calmette-Guerin (BCG) vaccine is primarily used against Mycobacterium tuberculosis (Mtb), although it has been shown to offer protection against other infectious diseases (Garly et al., 2003). Although BCG can train monocytes and macrophages *ex vivo*, it has been unclear how this vaccine is able to generate long-term innate immune memory. Recently, Kaufmann et al., showed that BCG reprograms HSCs and multipotent progenitors (MPPs) (Figure 1). Notably, this leads to the expansion and polarization of MPPs toward myelopoiesis. Further, the macrophages that arise from BCG-reprogrammed HSCs are epigenetically reprogrammed and greatly enhanced in their ability to offer protection to Mtb infection (Kaufmann et al., 2018). This infers that the BCG vaccination-induced epigenetic changes in HSCs and/or MPPs can be transferred to macrophages. This then enables macrophages to preserve a memory of exposure to pathogens, leading to an enhanced protection against infectious agents, such as Mtb.

Another recent study investigated the role of β-glucan in the training of HSCs (Mitroulis et al., 2018). The study showed that administration of β-glucan, a major component of the cell wall of *Candida albicans*, resulted in the expansion and increased transcription of innate immune regulators (e.g., IL-1β) in murine hematopoietic progenitors (Figure 1; Mitroulis et al., 2018). Furthermore, these β-glucan-mediated effects also offered enhanced responsiveness to a second lipopolysaccaride (LPS) challenge, as well as protection from chemotherapy-induced myelosuppression (Mitroulis et al., 2018). Therefore, it appears that β-glucan may reprogram HSCs and progenitors in a similar manner to that observed in BCG-trained HSCs (Kaufmann et al., 2018). Taken together these studies raise the following fascinating question: By what molecular mechanism is the reprogrammed transcriptional state in HSCs transmitted to their progeny?

## Trained Immunity is Regulated at the Epigenetic Level

In order to gain a deeper understanding of the mechanisms that regulate epigenetic memory, it is important to consider all factors that control gene regulation. DNA sequence alone is insufficient to predict whether any given gene will be transcribed. This complex regulation involves many factors which are ultimately encoded in chromatin-based modifications, including histone modifications. Critically, this suggests that in order for cells to accurately transmit a gene expression profile through multiple cell divisions, both the genetic and epigenetic code needs to be stably propagated to daughter cells.

The human genome contains ~3 billion bp, which is divided into 46 chromosomes in diploid cells. These chromosomes occupy discrete territories in the nucleus. Within these territories, ~147 bp of chromosomal DNA is wound around histone octamers which consist of two dimers of H3-H4 and H2A-H2B histone variants. These DNA-bound histones, or nucleosomes, can be recognized by proteins that harbor histone-binding domains which may either “read,” “write,” or “erase” epigenetic marks on the tails that extend out of the histone octamers (Zhang et al., 2015). Although many have been described, the most abundant and well-characterized histone modifications are methylation, acetylation, phosphorylation and ubiquitination. It appears that the combination of epigenetic modifications on nucleosomes dictate whether DNA exists in an accessible or inaccessible state. Yet, it remains unclear whether these epigenetic marks are causal to these chromatin states, or are simply a consequence of active or inactive chromatin.

Two well-studied groups of proteins that catalyse the epigenetic modifications of these two opposing chromatin states are the Trithorax (Trx) and Polycomb group (PcG) proteins (Geisler and Paro, 2015). Specifically, the Trx complex catalyses the deposition of the histone 3 lysine 4 trimethylation (H3K4me3), which is an active promoter chromatin mark. Mixed lineage Leukemia (MLL) is the human homolog of Trx. In contrast, the PcG proteins catalyse histone 3 lysine 27 (H3K27) methylation, which induces gene silencing. These epigenetic modifications, as well as many others, may ultimately determine whether genes will be expressed. Clearly, cells must have developed highly accurate regulatory mechanisms that are able to direct each complex to precise locations on the chromatin, at the correct time and in a context-specific manner.

**Figure 2:**
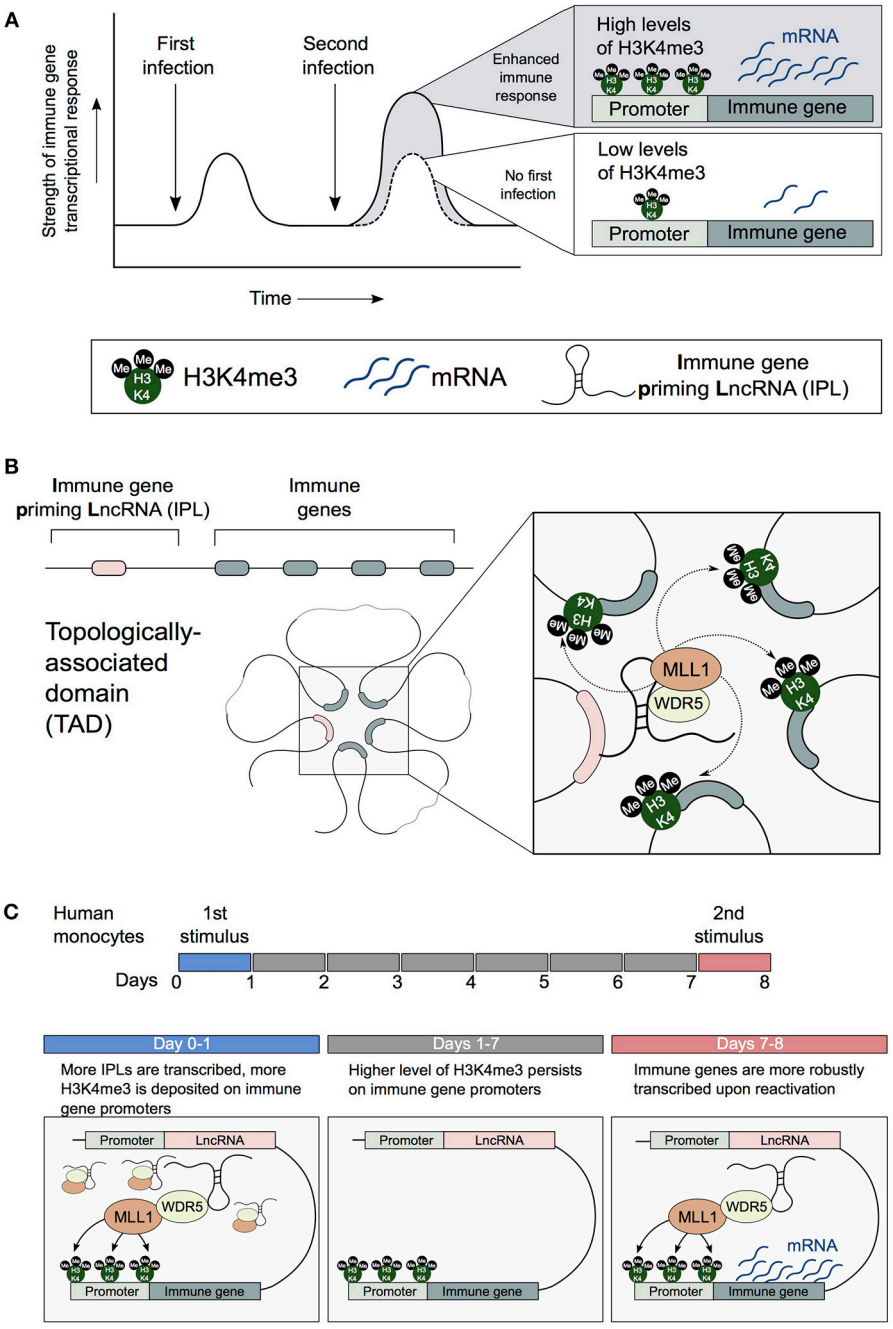
IPLs are central to the establishment of trained immune responses in monocytes. **(A)** Innate immune cells can develop enhanced resistance upon reinfection with the same or an unrelated pathogen. H3K4me3 accumulates on the promoters of trained innate immune genes. **(B)** 3D chromatin topology correctly positions immune priming lncRNAs (IPLs) proximal to innate immune gene promoters. This permits IPLs to direct the WDR5/MLL1 complex across immune gene promoters to facilitate their H3K4me3 epigenetic priming. **(C)** β-glucan upregulates IPLs, which leads to the deposition of H3K4me3 on immune gene promoters. As a result of the persistence of H3K4me3 chromatin marks, immune genes are more robustly transcribed upon reactivation by a secondary stimulus.

The enhanced immunity observed in trained monocytes is accompanied by the stable accumulation of epigenetic marks on dozens of innate immune responsive genes and enhancer elements. Specifically, there is the accumulation of the H3K4me3 promoter mark on immune gene promoters and the alteration of H3K4me1 and H3K27Ac epigenetic marks on enhancers (Quintin et al., 2012; Novakovic et al., 2016). H3K4me3 levels are directly correlated to transcriptional robustness, with higher promoter H3K4me3 levels associated with strong transcriptional responses that are uniform across cell populations (Lauberth et al., 2013). Therefore, as a consequence of β-glucan-mediated H3K4me3 epigenetic reprogramming, trained genes are more strongly and robustly transcribed (Figure 2A). How promoter and enhancer chromatin marks specifically accumulate on the promoters and enhancers of trained immune genes is poorly understood.

## Nuclear Architecture and Long Non-Coding RNAs (lncRNAs) Influence Epigenetic Gene Regulation

Chromatin 3D structure and chromosomal contacts have been shown to have a significant impact on gene regulation (Dixon et al., 2012; Fanucchi et al., 2013). Within the eukaryotic nucleus, DNA is folded into chromosomal loops allowing its compaction in 3D nuclear space. The development of the population-based chromosome conformation capture (3C) and derivative techniques [Hi-C,chromatin interaction analysis by paired-end tag sequencing (ChIA-PET) etc.] have revealed important insights into 3D genome architecture. Specifically, analyses of 3C-based data reveal that chromatin is divided into domains enriched in chromosomal looping contacts, referred to as topologically associating domains (TADs) (Dixon et al., 2012).

Within the DNA sequence there are various types of enhancer sequences that fine-tune gene expression. In certain circumstances, these regulatory sequences are located very far away from genes in one-dimensional space (even over 1 Mbp). However, because of the compaction and looping of DNA within TADs, these enhancer elements are brought into close proximity enabling them to contact gene promoters in three-dimensional space. Herein lies a more complex 3D framework to investigate genomic processes such as transcriptional regulation and epigenetic memory.

Enhancer-promoter chromosomal looping interactions are variable between different cell types and are critical to the establishment of cell-type specific gene expression profiles. Interestingly, these contacts may be pre-formed prior to gene expression, or be dynamically formed. An intriguing recent discovery showed that many TNF-responsive innate immune genes are located within the same TAD, and engaged in preformed chromosomal contact with enhancers located within the same TAD (Jin et al., 2013). This raises the questions of how these preformed DNA loops are maintained, and, how they may contribute to the epigenetic regulation of immune genes.

In addition to proteins such as transcription factors and epigenetic remodelers, long non-coding RNAs (lncRNAs) are emerging to be key modulators of gene activity by their influence upon epigenetic status. As a class, lncRNA transcripts are highly diverse, ranging from >200 nt to over 10 Kbp in length and may even be spliced and polyadenylated. Although not all lncRNAs are functional transcripts, several have been shown to tether protein-interacting partners near target genes to regulate their transcription as well as being able to act via other diverse mechanisms which have been extensively reviewed elsewhere (Engreitz et al., 2016; Magagula et al., 2017). Thus, despite lacking full protein coding potential, the activity of several lncRNAs have been shown to be associated with a multitude of disease states, such as cancer and inflammation (Gomez et al., 2013).

3D nuclear architecture can influence how lncRNA/protein complexes can access their target genes. Furthermore, lncRNA/protein complexes may work cooperatively with DNA elements to regulate the formation of enhancer and promoter looping interactions. A number of enhancer elements have been shown to be transcribed into lncRNAs, called enhancer RNAs (eRNAs). There is evidence that certain eRNAs interact with the Mediator complex to regulate the looping between enhancers and target gene promoters (Lai et al., 2013). However, many eRNAs may be byproducts that arise as a consequence of the accumulation of transcriptional regulators at enhancers. Thus it can be difficult to decrypt whether lncRNAs are indeed functional transcripts, or whether they simply are the result of mere transcriptional activity at the lncRNA promoter, or lncRNA transcription/splicing events (Engreitz et al., 2017). Nonetheless, several carefully conducted studies have revealed that dozens of lncRNAs are indeed functional transcripts. For example, using both loss and gain-of-function approaches, lincRNA-Cox2 has been shown to interact with hnRNPA2/B1 and hnRNP-A/B to repress a large number of immune genes (Carpenter et al., 2013). Furthermore, HOXA distal transcript antisense RNA (HOTTIP) and NeST are two lncRNAs that have been shown to directly interact with WD repeat-containing protein 5 (WDR5) to direct mixed lineage leukemia protein 1 (MLL1) to target genes, to catalyse the trimethylation of H3K4me3 at target gene promoters (Wang et al., 2011; Gomez et al., 2013). However, even though several lncRNAs have been established to direct histone modifying enzymes to target genes, the role of lncRNAs in directly regulating trained immune responses remains poorly explored.

## Long Non-Coding RNAs (lncRNAs) Regulate the Epigenetic Reprogramming of Innate Immune Genes

Recently we investigated how the combined influence of 3D chromatin topology and lncRNA-related regulation impacts the H3K4me3-primed state of trained innate immune gene promoters. We showed that 3D chromatin topology enables key trained immune genes (e.g., IL1β, IL-6, IL8) to engage in chromosomal contacts with a newly identified subset of lncRNAs. We term these lncRNAs immune-gene priming lncRNAs (IPLs) (Figure 2B; Fanucchi et al., 2018). From our candidate IPLs, we characterized one lncRNA, which we have named UMLILO (Upstream Master LncRNA of the Inflammatory chemokine LOcus). This IPL formed chromosomal contacts with the ELR+ CXCL chemokines (IL-8, CXCL1, CXCL2, and CXCL3). We demonstrated that UMLILO acts in *cis* to direct the WDR5/MLL1 complex across the CXCL chemokine promoters enabling their H3K4me3 epigenetic priming, prior to their transcriptional activation. Other trained immune genes (e.g., IL-6 and IL1β) are also regulated in a similar IPL-mediated manner. Importantly, β-glucan-induced training of monocytes resulted in an NFAT (Nuclear Factor of Activated T cells)-mediated increase in transcription of IPLs, which in turn resulted in the epigenetic reprogramming of the innate immune genes (Figure 2C). This study provides the first evidence that lncRNA-mediated regulation is central to the establishment of trained immunity.

## Do IPLs Regulate Other Aspects of Trained Immunity?

**Figure 3:**
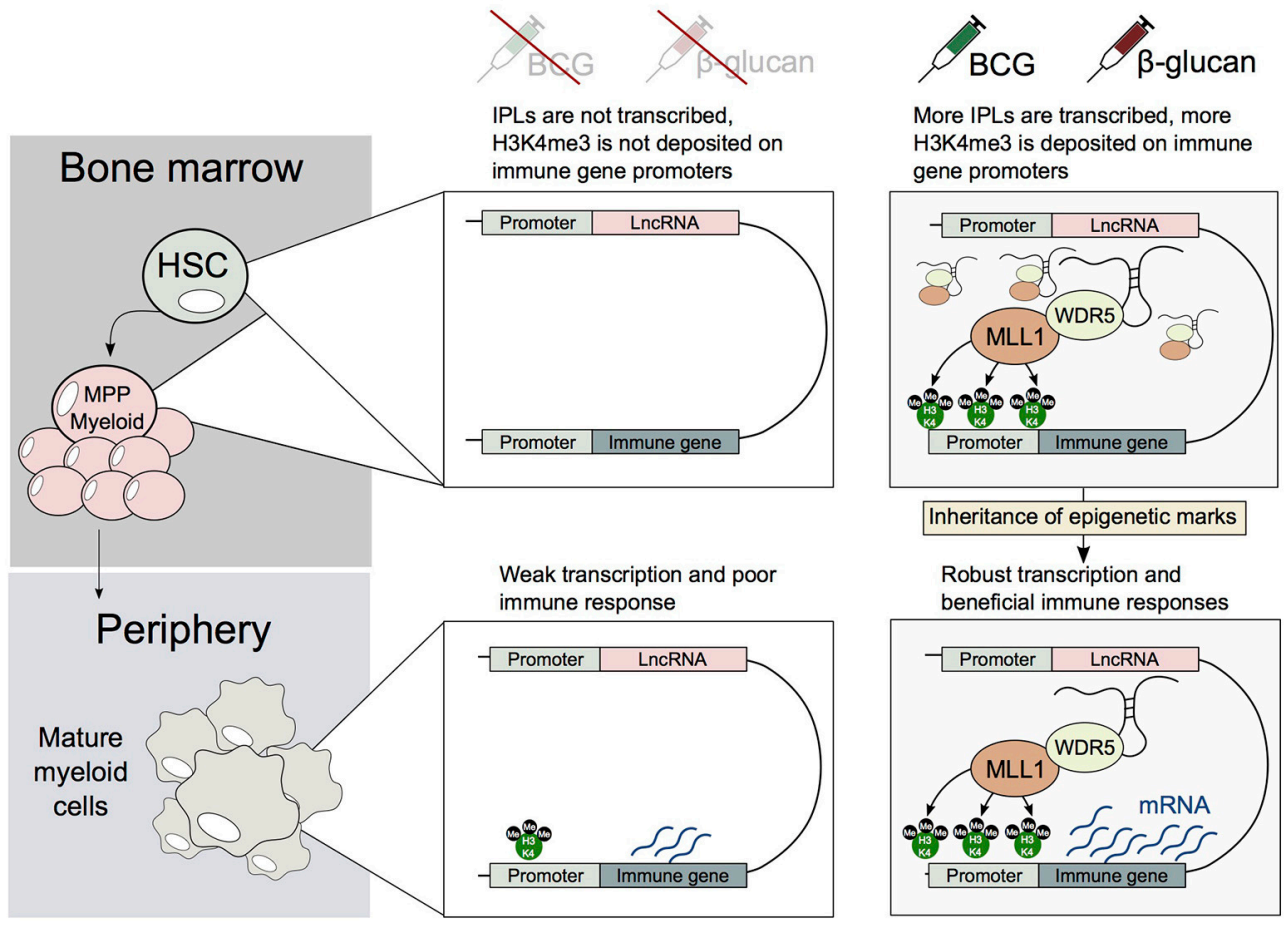
A potential mechanism describing how HSCs and/or MPPs trained by BCG or β-glucan may transmit their epigenetic memory to myeloid cells. Upon exposure to BCG or β-glucan, HSCs and/or MPPs upregulate IPLs, which in turn leads to the accumulation of H3K4me3 marks on immune genes. This epigenetic memory is then transmitted to mature myeloid cells, leading the generation of trained monocytes and macrophages.

Bone marrow-derived macrophages (BMDMs) derived from BCG vaccinated mice have been shown to exist in an epigenetically primed state prior to Mtb infection, and possess an enhanced ability to control Mtb infection (Kaufmann et al., 2018). This implies that HSCs or MPPs may be epigenetically reprogrammed by BCG, and this altered epigenetic state is transmitted to their progeny. Yet it remains unknown when and how this epigenetic state is established. We speculate that HSCs may be trained in an IPL-dependent manner (Figure 3). Specifically, in a similar manner to that we observed in primary circulating monocytes, β-glucan, or BCG may induce a transcriptional program, that would lead to the upregulation of IPLs in HSCs and/or MPPs. These pathways may also involve NFAT-mediated signaling, as well as other myeloid specific transcription factors such as PU.1 and CCAAT-enhancer binding proteins (C/EBPβ). This is in turn would direct the WDR5/MLL1 complex to innate immune promoters, to facilitate their H3K4me3 epigenetic priming in HSCs. These epigenetic changes would then need to be transmitted from HSCs and/or progenitors to the mature myeloid cells (e.g., macrophages). Accordingly, identifying the repertoire of lncRNAs involved in “educating” HSCs may provide novel approaches to develop better vaccines to target Mtb as well as other infectious diseases.

In contrast to circulating monocytes, tissue-resident macrophages such as alveolar macrophages (AM) have been shown to self-maintain throughout the lifespan of an organism and are maintained independently of circulating monocytes (Hashimoto et al., 2013). Although, the generation of immune memory in tissue-resident innate immune cells is poorly investigated, a recent study revealed that AM develop innate immune memory which protects the host from a subsequent lethal dose of pneumonia (Yao et al., 2018). This occurred in a manner that did not involve bone marrow progenitors or circulating monocytes (Yao et al., 2018). Interestingly, this study revealed that CD8+ T cells are required to prime AM via an IFNγ-dependent mechanism. Although the authors did not investigate whether the AM were epigenetically reprogrammed, it seems reasonable to speculate that IPLs regulate AM-associated innate immune memory, in a manner similar to that observed in circulating monocytes. Furthermore, as NeST is a WDR5-interacting lncRNA that is induced by IFNγ (Gomez et al., 2013), it is possible that IFNγ-dependent signaling activates an entirely new set of lncRNAs with “IPL-like” properties.

## The Therapeutic Potential of IPLs

Trained immune responses have been shown to be aberrantly regulated in inflammatory disease states. Therefore, inducers of trained immunity could be used to reverse immunotolerant states, such as sepsis or cancer, or alternatively as a protective therapeutic approach to prevent infections, such as vaccination (recently reviewed in Mourits et al., 2018). For example, BCG is already administered to patients who are being treated for bladder cancer (Kawai et al., 2013). Clearly, targeting specific aspects of trained immunity may represent a powerful therapeutic approach to target diseases with an inflammatory basis.

As epigenetic reprogramming of immune genes is the underlying mechanism of trained immunity, targeting epigenetic modifiers represents a novel approach to alter immune gene expression in different inflammatory conditions. However, many inhibitors of epigenetic regulators affect numerous genes, and therefore may exhibit broad off target effects. As a result, there is a great need to develop highly specific small molecule inhibitors that are able to more discreetly influence immune-gene epigenetic regulation. As the IPLs regulate the epigenetic priming of immune genes during innate immune responses, they represent an entirely new class of druggable targets that may represent a valuable therapeutic strategy in achieving tailored immunomodulation.

RNA sequencing performed on unstimulated monocytes that were trained with β-glucan did not reveal major differences in the expression of trained immune genes (Saeed et al., 2014; Novakovic et al., 2016). This reveals that trained monocytes in a resting state are epigenetically primed, but do not display significant changes in the transcription of immune genes such as IL-6, IL1β, CCL2, and IL-8. As IPLs are expressed at very low levels (a few copies per cell), they are not readily detected in these RNAseq datasets. However, as IPLs coordinate the H3K4me3 deposition of trained immune genes, they are expressed prior to the transcriptional activation of the trained immune genes. Importantly, the level of IPL expression determines H3K4me3 levels (a correlate for the “degree” of training). This indicates that assaying IPL transcription levels using more sensitive molecular biology tools may be a useful biomarker for assessing effective innate immune memory and activation.

## Conclusions and Future Perspectives

The discovery that lncRNAs and 3D nuclear architecture regulate the epigenetic training of immune cells reveals that these are two critical factors involved in the establishment and maintenance of epigenetic memory. It is clear that future studies will need to consider both of these factors in order to address the precise mechanism of how epigenetic memory is transmitted during trained immune responses. Further, future studies will be required to investigate the efficacy of drugging lncRNAs in the context of trained immune responses.

## Author Contributions

All authors listed have made a substantial, direct and intellectual contribution to the work, and approved it for publication.

### Conflict of Interest Statement

The authors declare that the research was conducted in the absence of any commercial or financial relationships that could be construed as a potential conflict of interest.
